# Egg covering in cavity nesting birds may prevent nest usurpation by other species

**DOI:** 10.1007/s00265-021-03045-w

**Published:** 2021-07-31

**Authors:** Tore Slagsvold, Karen L. Wiebe

**Affiliations:** 1 Centre for Ecological and Evolutionary Synthesis (CEES), Department of Biosciences, University of Oslo, N-0316 Oslo, Norway; 2 Department of Biology, University of Saskatchewan, Saskatoon, Canada

**Keywords:** Bird nests, Egg covering, Nest lining, Nest materials, *Ficedula Parus*

## Abstract

**Abstract:**

Some birds cover their eggs with nest material when they leave to forage. It has been suggested that such egg-covering aids thermoregulation or prevents predation but here we present a new hypothesis, that secondary cavity-nesting species cover their eggs to prevent nest usurpation by other birds. When the bottom of the cavity is dark, as when eggs are covered by nest material, it may be difficult for a prospecting competitor to see whether a defending nest owner or a predator is hiding inside the cavity. Competitors may therefore hesitate to enter dark cavities. We filmed 21 great tit (*Parus major*) nests during the egg-laying period and found that the female spent bouts of highly variable length outside the nest box (range 0.3–250 min, *n* = 51), so prospecting small passerines would have difficulty predicting whether an aggressive tit owner was in the box or would soon return. We presented prospecting male pied flycatchers (*Ficedula hypoleuca*) with a dyad of boxes (*n* = 93), each containing a great tit nest but only one with visible eggs. Flycatchers hesitated more to enter a nest box with no visible tit eggs than a box with exposed eggs. This was most evident for nest boxes with dark versus light interior paint, supporting the idea that better interior illumination makes prospecting birds more confident about entering an unfamiliar cavity. The usurpation and predation hypotheses are not mutually exclusive because both competitors and small predators may hesitate to enter dark, enclosed spaces if visibility is low.

**Significance statement:**

Some birds deposit a layer of material on top of the eggs when they leave the nest. Several hypotheses have been proposed for such egg covering, for example that it may insulate the eggs and reduce the risk of nest predation. We propose a new hypothesis, namely that secondary hole-nesting birds cover their eggs when they leave the nest to prevent usurpation of the cavity by other birds. Great tits that we filmed at the nest during the egg-laying period could be absent for long periods. To test the hypothesis, we presented male pied flycatchers, potential nest competitors, with a dyad of nest boxes, each containing a great tit nest but only one with visible tit eggs. In support of the prediction, prospecting flycatchers hesitated to enter dark cavities with dark floors relative to boxes with exposed, reflective eggs.

## Introduction

Most birds build a nest to contain their eggs and offspring and this may provide several benefits including thermoregulation, reducing predation risk and attracting a mate (Hansell 2000; Gould and Gould 2007). In addition to the base layers supporting the eggs, some birds deposit a layer of material on top of the eggs when they leave the nest during the egg-laying period, or during incubation (Collias and Collias 1984; Prokop and Trnka 2011). In secondary hole-nesting birds, egg covering has been studied in most detail in parids (Haftorn and Slagsvold 1995; White and Kennedy 1997). In the egg-laying period, the female typically covers the eggs with a mat of hairs and fur in the morning when leaving the nest and uncovers them when she returns in the evening to roost and to lay another egg the next morning.

Several hypotheses have been proposed for egg covering and these are not mutually exclusive. Perhaps the oldest ideas are that egg cover reduces heat loss from the nest and also reduces nest predation by hiding eggs from predators (Collias and Collias 1984). In open-nesting species such as mallards (*Anas plathyrhynchos*), the cryptic value of nest cover seems apparent (Kreisinger and Albrecht 2008). Predators inspecting a cavity nest from the entrance might be similarly deceived into perceiving the cavity is empty and not enter (Haftorn and Slagsvold 1995). Whatever its function, it takes time and energy for a parent to collect the material and to cover and uncover eggs. For instance, in great tits (*Parus major*), it took 4–15 min whenever the eggs were covered or uncovered (Haftorn and Slagsvold 1995). In a comparative study of great tits across Europe, a positive correlation was found between extent of egg covering and predation risk, and a negative correlation between egg covering and ambient temperatures (Loukola et al. 2020) suggesting that birds invest more in covering when environmental pressures warrant it.

Egg covering may play a role in competition among species of secondary cavity-nesting birds. For instance, when exposed to playback song of a male (pied flycatcher) *Ficedula hypoleuca* in the egg-laying period, great tits added more lining materials on top of their eggs than control birds (Loukola et al. 2014a) and the extent of egg covering was positively correlated with the local population density of pied or collared (*F. albicollis*) flycatchers in the comparative study across Europe (Loukola et al. 2020). It was suggested that the tits add the cover to prevent prospecting flycatchers from assessing the clutch size, presuming that migratory flycatchers use such information of a resident species when deciding whether or not to settle in the vicinity and use a similar-looking nest cavity (Loukola et al. 2013, 2020). Here we present a new hypothesis to explain such findings and propose that egg covering by tits reduces the risk of cavity usurpation by other birds, and in particular by *Ficedula* flycatchers in Europe.

The usurpation hypothesis is based on findings in North America that egg covering in black-capped chickadees (*Poecile atricapillus*), and in tufted titmice (*Baeolophus bicolour*), reduced the damage of their nests by usurping house wrens (*Troglodytes aedon*) (White and Kennedy 1997). We suggest that the egg covering discourages prospecting birds from entering unfamiliar nest holes because material over the white eggs keeps the cavity bottom dark and hard to see. A dark floor of a cavity could be perceived as dangerous because it might be concealing an aggressive, incubating tit owner or a predator. On the other hand, exposed whitish eggs illuminate the floor of the nest cavity, making it easy for a prospecting bird to see from the cavity entrance that there is no occupant currently in the space. The laying period of tits is relatively long, often between 7 and 10 days, and because the parents may forage away from the nest during this time, the cavity may be exposed to visits by prospecting birds, like flycatchers.

Great tits are resident whereas pied flycatchers are long-distant migrants arriving in late spring when it may be difficult to find a suitable nest cavity (Dale et al. 1992). Pied flycatchers often prefer nest boxes containing old nest materials compared to empty boxes probably to save costs of nest building (Orell et al. 1993; Loukola et al. 2014b). Upon discovering an unoccupied box, a flycatcher may perceive itself as the owner within a few hours and attract a female that may start nest building quickly, often on the same day (Dale and Slagsvold 1995, 1996). Although a flycatcher (~ 12 g) is smaller than a great tit (~ 17 g), it will not give up a nest cavity easily once it perceives itself as the owner. However, if both species enter a cavity, the great tit frequently kills the flycatcher (Slagsvold 1975; Merilä and Wiggins 1995; Samplonius and Both 2019). A great tit may not only attack prospecting flycatchers near its own nest but may also defend empty nest cavities elsewhere on its territory (Slagsvold and Wiebe 2020). However, chasing intruders is costly and avoiding fights in an enclosed cavity obviously reduces the risk of injury to the owner and to the eggs, selecting for alternate ways to avoid nest usurpation. The tit may abandon the cavity if the flycatcher is killed inside (Ahola et al. 2007).

Here we first recorded the length of time female great tits were naturally away from their nests during the egg-laying period to study whether prospecting pied flycatchers have enough time to inspect the cavity before the owner returns. Second, we manipulated features of nest boxes to study whether box depth (small, medium, or large), and a light or dark interior color, caused prospecting flycatchers to delay their entrance. Using a pairwise experimental design, we offered flycatchers choices between a dyad of nest boxes of the same size and interior color. In the first treatment, flycatchers chose between a box with an empty tit nest cup and a box with a tit nest with uncovered eggs. We predicted that if a clutch of eggs signals to the flycatcher that the cavity is occupied and unavailable, it would avoid entering the box with visible eggs and, if so, this would be evidence against the usurpation hypothesis. Finally, the most critical test of the hypothesis was to let the flycatchers choose between two nest boxes that both contained a tit nest with eggs but where one nest had uncovered eggs and the other box had eggs covered by lining material. Here, we predicted that the flycatchers should more readily enter the box with uncovered eggs and in particular, the degree of preference for the box with exposed eggs should be greater when both boxes of a dyad had a dark-painted interior. However, we expected the degree of preference to be highest for medium-sized nest boxes because with small, shallow cavities it may be easy for a prospecting bird to see the inside of the box irrespective of bottom illumination whereas with large, deep nest cavities, it may be difficult to see the bottom from the entrance hole, whatever the contents.

## Methods

### Study area and study species

The study was conducted during 2016–2018 in woodlands with mixed deciduous and coniferous trees at Dæli (59°56’N, 10°32’E) and Brenna (60°01’N, 10°38’E) near Oslo, Norway. The woodlands are managed and contain few natural cavities. The wooden nest boxes were attached to live trees about 1.5 m above the ground, and had a 32-mm diameter entrance hole and a wall thickness of about 21 mm. Here the pied flycatchers arrive from spring migration in late April and first half of May. Males arrive a few days before females and only those males which secure a suitable nest site may attract a mate (Dale and Slagsvold 1996). In pied flycatchers and great tits, only the female builds the nest and incubates. To construct a nest inside the box, tits use lining materials of fur, hair and sometimes feathers on top of a thick layer of moss (Aasen and Slagsvold 2020), whereas flycatchers use mainly dead and dry leaves and straw and thin bark from trees (Lundberg and Alatalo 1992).

### Box attentiveness by tits

To record behavior in absence of human disturbance, we used digital camcorders with 32 × optical zoom, on tripods placed 5–8 m from focal nest boxes of the tits and flycatchers. During 1–31 May in 2016 and 2017, we filmed 21 great tit nests in the egg-laying period when 3–10 eggs had been laid. All nests were first breeding attempts of the season and were filmed only once, for 2.9–5.6 h between 0705–1225 h of the day. The durations of ´on´ and ´off´ bouts of the female tit were calculated using only periods when the exact start and stop of the respective bout was known. However, to avoid underestimation, we used the duration of the whole filming for two nests where the female never entered, and for three nests with only one entry each, we used the duration of the longest period off the nest.

### Experimental design

To study cavity inspection by male pied flycatchers, we used a pairwise design, offering two nest boxes with different content in each trial (Table 1). Using pairwise tests, the effects of most confounding variables could be controlled, including type of habitat, time of day, condition of the focal bird, motivation, and weather conditions. All boxes offered contained a freshly built great tit nest in order to minimize the number of fleas which might have confounded flycatcher choices. The tit nests were collected from other distant boxes to which we then added old nest material instead. The four great tit eggs put in a nest box (half the average clutch size in our area) were chosen at random from a sample of 10–30 eggs each time. The eggs were collected by removing only 1–4 eggs from great tit nests to avoid desertion. The trials were conducted during 3 May-7 June in 2016–2018. The dyad was filmed only once for 0.7–6.1 h (total 264 h of filming) between 0620 and 1520 h of the day, ensuring that both boxes were within the field of view.

**Table. 1:** Pairwise trials to study nest box inspection by prospecting male pied flycatchers. Each nest box of a dyad held a great tit nest, either with four great tit eggs without a lining cover (Box A), or with four covered eggs or an open nest cup without eggs (Box B)

Experiment	Nest box size	Interior color of nest box	No. of trials	Content of nest boxes in the dyad	
				Box A	Box B
1	Small	Unpainted	20	Uncovered eggs	Covered eggs
2	Medium	Light-painted	12	Uncovered eggs	Covered eggs
3	Medium	Light-painted	14	Uncovered eggs	Open cup, no eggs
4	Medium	Dark-painted	13	Uncovered eggs	Covered eggs
5	Medium	Dark-painted	12	Uncovered eggs	Open cup, no eggs
6	Large	Unpainted	22	Uncovered eggs	Covered eggs

We selected male pied flycatchers that had settled at a nest box and that were unmated and singing to attract a female. Just before the start of a trial, the initial empty nest box was blocked and two nest boxes were erected on trees 4–10 m away and 2–5 m apart. Short distances were used so that the focal male would rapidly discover the new nest boxes. Only a few of the flycatchers had been ringed previously but we assumed that the males filmed for each trial were different based on their spatial distribution, simultaneous singing, and the dorsal color and size of the white forehead patch.

**Fig. 1:**
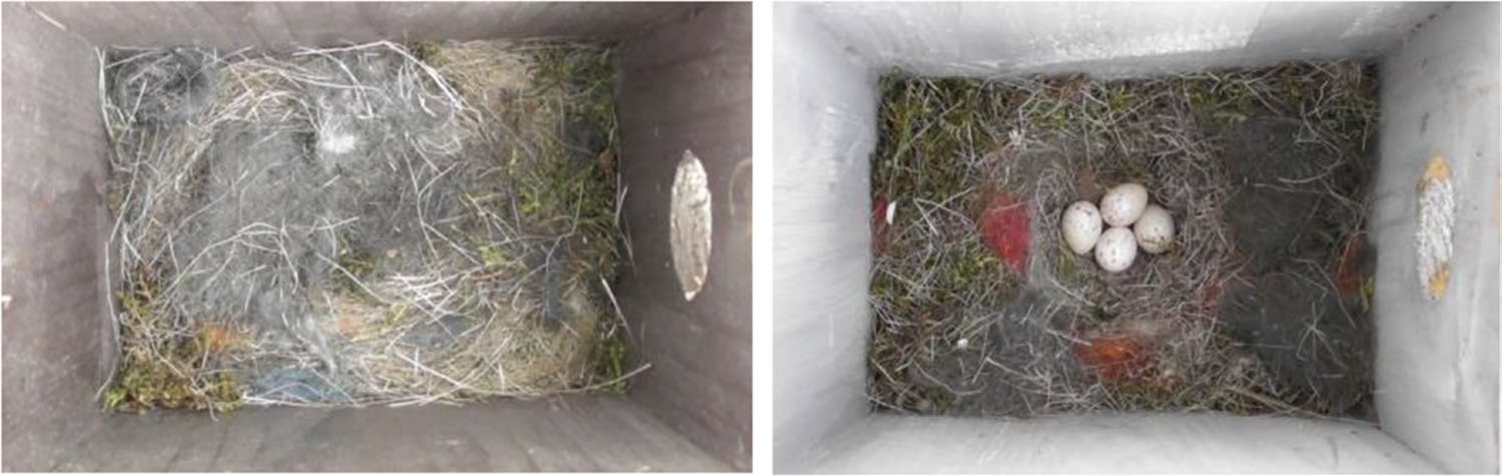
Pied flycatchers hesitated longer to enter an unfamiliar nest box with dark interior paint and great tit eggs covered with lining materials (left) than a box with light interior paint and four exposed tit eggs (right)

We used boxes of three sizes (Table 1); bottom area and distance from entrance hole to the bottom: small (100 cm^2^; 12 cm), medium-sized (150 cm^2^; 14 cm), or large (155 cm^2^; 24 cm). The depth of the fitted tit nests (from the nest rim to the box floor) was 8 cm for the small boxes, 9 cm for medium, and 11 cm for large. Half the medium-sized nest boxes were painted light brown-grey inside and half were painted dark brown-grey (Table 1; Fig. 1). The small and large boxes used had hung in the forest for at least a couple of years, and the darkness of their unpainted wooden interior was intermediate to the painted ones. Both boxes in a pair were always of the same size and color, and whether or not it contained four uncovered eggs was chosen at random, as was also which box should be erected to the right and left.

The trials with medium-sized nest boxes were of two types (Table 1); the nest box with four exposed tit eggs was either in a dyad with a nest box containing four covered tit eggs (experiments 2 and 4), or a box with an empty tit nest (experiments 3 and 5). The results were similar and therefore combined if not otherwise stated. The lining material put on top of eggs was exchanged between trials and was only thick enough to just cover the eggs. The particular nest boxes to be used for a trial were chosen randomly from a pool of 15 small, 12 medium-sized, and 15 large boxes. If prospecting females are reluctant to enter a dark nest, it may pay the male to remove the cover and prepare the nest cup for nest building. Hence, at the end of trials, we recorded whether the male had removed or arranged any lining materials before we took down the paired boxes and reopened the original box.

### Statistical analyses

It was not possible to record data blind because our study involved focal animals in the field. We defined a nest box visit as when a bird perched at the entrance hole, whether or not it entered the box. We only used trials in which the male had visited the entrance hole of both nest boxes of a dyad and had entered at least once by the time the film ran out, leaving 93 of 104 trials. When a male had entered only one box, we assumed (conservatively) that he would have entered the other box when the filming ended. This occurred in two, seven, and six trials with small, medium and large nest boxes, respectively and was not biased by box contents, occurring seven times for the box with uncovered eggs, and eight for the alternate box. We included such cases because they are still informative about hesitancy to enter a type of nest box. Results were similar if such cases were excluded or not.

The male flycatcher had to visit a box to assess its interior quality. Therefore, we expected that the elapsed time between the start of filming and the first visit to any box of a dyad would not differ according to box contents and this was confirmed (paired *t*-test, *t* = 0.62, df = 91, *p* = 0.54). The box with uncovered eggs was visited first in 44 cases and the alternate box in 49 cases.

We studied whether a focal male flycatcher preferred a nest box of a dyad (1) by recording the number visits to a nest box before the first entry of the same box, and (2) by recording the time elapsing from the first visit of the box and the first entry of the same box. We assumed that it was mainly the number of visits (i.e., quick glances from the entrance) that helped to inform the male of box contents, whereas the time between visits may have been affected by random disturbances occurring outside the box. For example, a passing predator might cause a prospecting bird to wait to check the box again until the ´coast is clear´, and cause the length of time to enter a box to increase in a way that is not related to its interior contents. Therefore, we assumed that our first variable would best reflect hesitancy to enter. Obviously, the two variables were correlated (e.g., Spearman rank correlation, *r*_s_ = 0.75, *n* = 93, *p* < 0.001, between the number of visits before the entry of any nest box of a dyad and the time elapsing from the first visit to the first entry of any box). However, we also present data for the second variable because it is the time delay of flycatcher entry that matters for how long the tits can be away from their nest cavity. As a measure of male motivation or popularity of a nest box after inspection, we calculated the frequency of visits based on the total number of visits to a nest box divided by the length of time elapsing from when both boxes of a dyad had been visited and the end of filming.

The male pied flycatchers were unmated and were trying to attract a female so we studied whether appearing females responded to box contents when assessing nest site quality. However, the prospecting behavior of females is strongly affected by male behavior; when a female appears, the male enters the nest cavity first and makes enticement calls to stimulate her to follow (Lundberg and Alatalo 1992). A female flycatcher appeared during only 18 of the video-filmed trials (19%, *n* = 93), which did not allow analyses with regard to nest box size and interior color. When the female only visited one of the nest boxes of a dyad (*n* = 4) again we assigned conservatively a visitation time to the other box equal to the end of the trial.

In male flycatchers, the time elapsing until the first visit of any nest box of a dyad, and the visiting rate after both boxes had been visited, were log-transformed to achieve normality and we used *t*-tests and ANOVA. The means presented are those after back-transformation. The number of visits by the focal male to the nest boxes before the first entry of the same box, and the time elapsing from the first visit of a nest box to the first entry of the same box, could not be transformed for normality. Neither could the time to a first visit to a box by a female be transformed so for these variables we used nonparametric Kruskal–Wallis, Mann–Whitney *U*, or Wilcoxon paired signed-ranks tests. Statistical tests are two-tailed with an *α*-level of 0.05.

## Results

### Visits by tits and flycatchers of tit nests

**Fig. 2:**
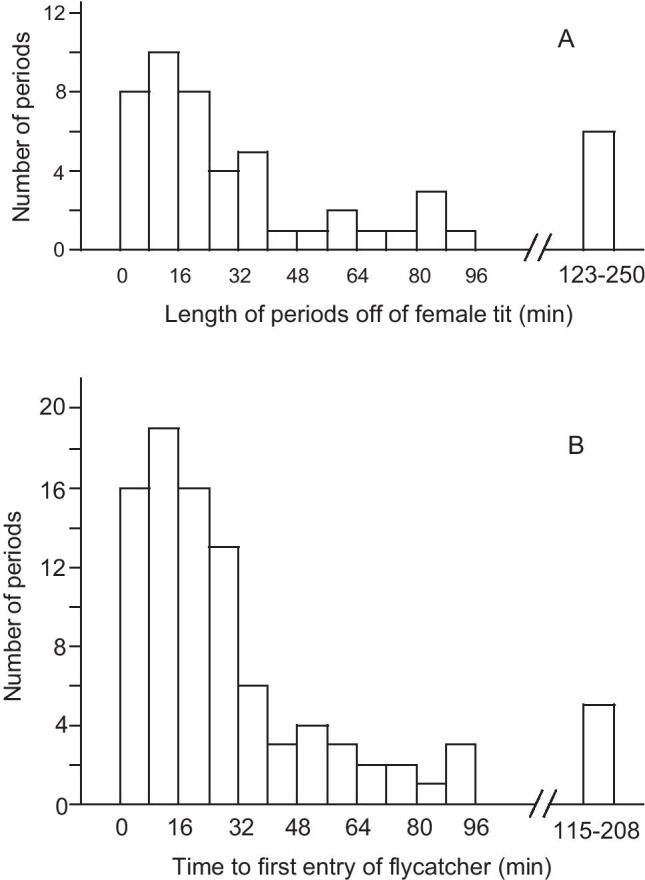
(**A**) Frequency of the length of periods (*n* = 51) off the nest of female great tits video filmed in the egg-laying period. Data from 21 nests filmed for a total of 78 h. (**B**) Frequency of length of periods lasting from start of video filming to the focal male pied flycatcher entered for the first time a trial box that contained a great tit nest. Data from 93 trials

During the laying period, 19 of 21 female great tits entered their nest boxes during filming, spending 0.2–57 min inside (median 46 s, *n* = 62). Females spent most of the time outside the box (median 98.5%, range 54–100%), for periods of 0.3–250 min (median 23 min, *n* = 51; Fig. 2A). When it was possible to see from the films, females brought nest materials 81% (*n* = 36) of the times they entered. Six of the male great tits visited the nest box, and five pied flycatchers (four males and one female) appeared at the tit box; three males perched at the box opening and only one entered the box. No box was usurped by flycatchers.

### Visits by male flycatchers to dyads of nests boxes

**Fig. 3:**
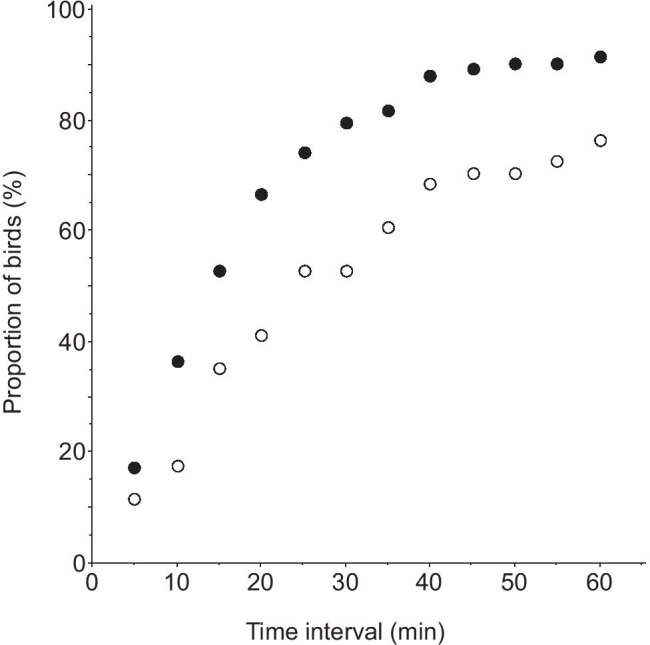
Cumulative distribution for the duration of absence of the female great tit from her nest box during the egg-laying period (open symbols, *n* = 51), and the time elapsing before the first visit of a focal male pied flycatcher to any nest box of a dyad during trials where both boxes offered contained a great tit nest (filled symbols, *n* = 93)

Usually, the unmated focal male flycatcher visited the boxes in the dyad several times before entering one. The first visit to a trial nest box occurred 0.8–158 min after the filming started (median 14 min), and the male entered the first box of the dyad after 2.3–208 min (median 21 min, Fig. 2B). They made 1–26 visits (median 2) before entering any box, spending 0.2–141 min (median 1.5 min) from the first visit to the first entry, and visiting the boxes 2–113 times (median 21) in total during filming. A comparison of the length of time it took for a male flycatcher to first enter an experimental nest box with the time female great tits were outside their nest box showed that after 23 min (the median time of great tit absence from her nest, Fig. 3), 70% (*n* = 93) of the male flycatchers had entered at least one of the experimental boxes. When 60 min had elapsed, 91% (*n* = 93) of the males had done so.

The total number of nest box visits of a prospecting male flycatcher from the time filming started until the first entry of any nest box of a dyad did not differ between small, medium-sized (data from experiments 2–5 combined), and large nest boxes (Kruskal–Wallis test, *n* = 93, *H* = 0.68, *p* = 0.71). Neither was the time elapsing from the first visit of any nest box and the first entry associated with box size (*n* = 93, *H* = 2.90, *p* = 0.23). However, after the male had visited both boxes in a dyad, his subsequent visiting rate differed strongly according to nest box size (ANOVA, *F*_2,90_ = 12.47, *p* < 0.001), being lower for small and large nest boxes than for medium-sized ones.

### Effect of nest contents and painting

**Fig. 4:**
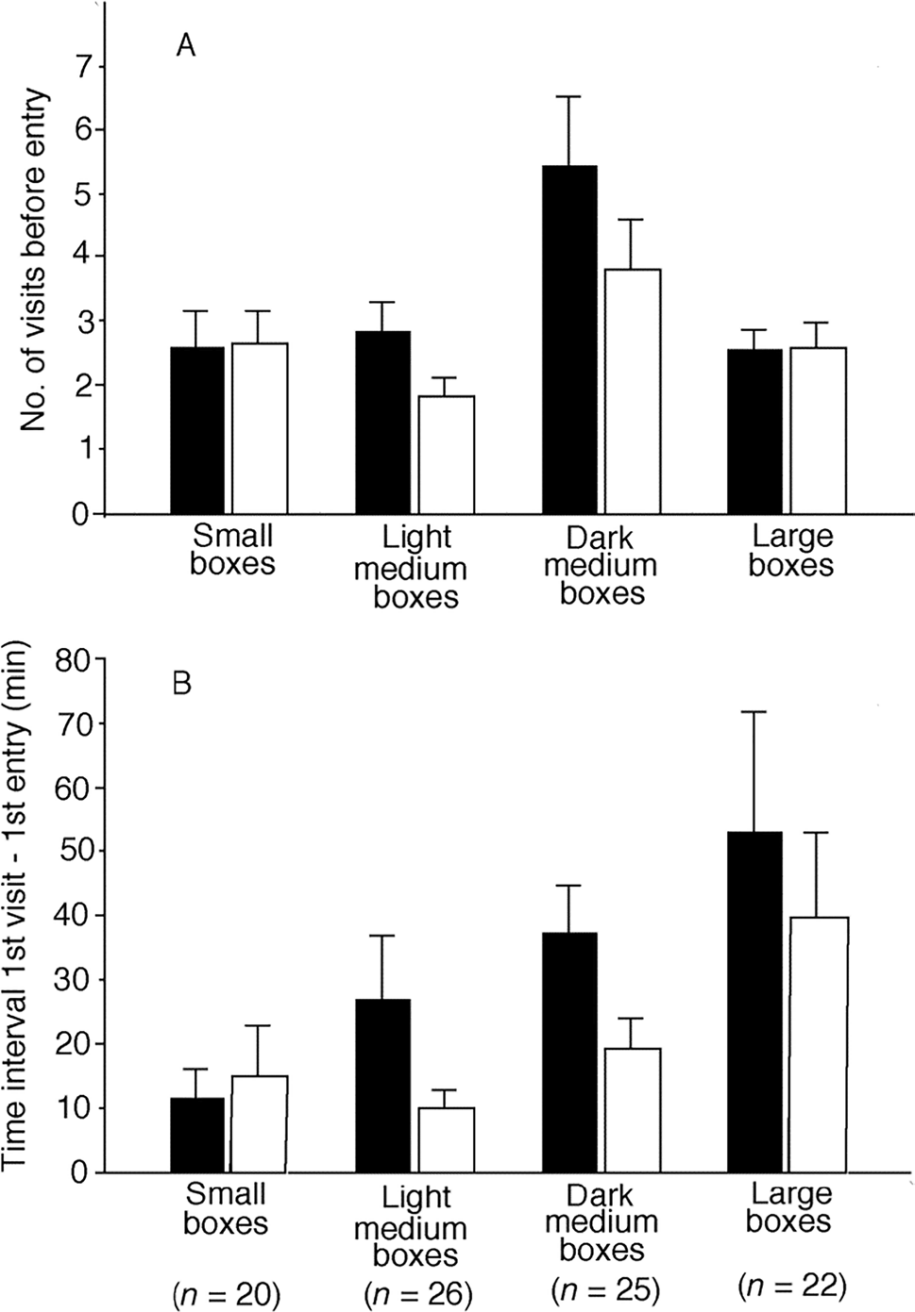
Nest box visiting behavior of male pied flycatchers, showing mean values (+ SE) for **A** the number of visits to each nest box before entry, and **B** the time elapsing from the first visit of a focal nest box to the first entry of the same box, for various types of nest boxes that all contained a great tit nest. The interior of medium-sized nest boxes was either light or dark painted. The trials were pairwise, where one nest box had no visible tit eggs (empty nest cup or four covered tit eggs; filled columns), and the other nest box had four uncovered great tit eggs (open columns). The number of trials is shown below the lower graph

The number of visits before the first entry by the male flycatcher was lower for the nest box with uncovered eggs than for the alternative nest box of a dyad (Wilcoxon paired signed-ranks test, *z* =  − 2.64, *n* = 93, *p* = 0.008; Fig. 4A). Also, the time elapsing from the first visit of a nest box to the first entry of the same box was significantly shorter for the box with uncovered eggs than the alternate box (*z* =  − 2.08, *n* = 93, *p* = 0.038; Fig. 4B). The flycatcher’s tendency to enter boxes with exposed eggs depended on box size. The difference in visitation rate between the boxes of a dyad was significant for medium-sized nest boxes (*z* =  − 3.37, *n* = 51, *p* < 0.001) but not for small or large ones (*p* > 0.83, tests not shown; Fig. 4A). Similarly, the shorter time to enter a box with exposed eggs relative to the alternate box in the dyad was significant for medium-sized ones (*z* =  − 2.40, *n* = 51, *p* = 0.016) but not for small or large nest boxes (*p* > 0.50, tests not shown; Fig. 4B).

Holding box size constant, i.e., within medium-sized nest boxes, we then tested whether the tendency of flycatchers to enter the box with exposed eggs relatively quickly compared to the other box in the dyad depended on the interior illumination of the box. The flycatchers made fewer visits before they entered the nest box of a dyad with exposed eggs, both when the alternate box held tit eggs covered with nest materials (experiment 2 and 4; *z* = -2.40, *n* = 25, *p* = 0.017), and when the alternate box had an empty tit nest (experiments 3 and 5; Wilcoxon paired signed ranks test, *z* =  − 2.36, *n* = 26, *p* = 0.018). There was a non-significant trend that the time from the first visit of a nest box to the first entry of the same box was shorter for the box in the dyad with exposed eggs than the alternate box (eggs covered: *z* =  − 1.87, *n* = 25, *p* = 0.062; empty tit nest: *z* =  − 1.40, *n* = 26, *p* = 0.16).

Male flycatchers made fewer visits before entering a medium-sized box with exposed eggs relative to the other box in the dyad both for light-painted boxes (experiments 2 and 3; Wilcoxon paired signed-ranks test, *z* =  − 2.28, *n* = 26, *p* = 0.023), and dark-painted boxes (experiments 4 and 5; *z* =  − 2.50, *n* = 25, *p* = 0.012). The mean value was lowest for the light boxes with uncovered eggs (1.85 visits), and greatest for the dark boxes with no visible tit eggs (5.40 visits; Fig. 4A). Similarly, there was a non-significant trend that the time it took to enter a box was shorter when the data were split into light-painted boxes (*z* =  − 1.87, *n* = 26, *p* = 0.062), and dark-painted boxes (*z* =  − 1.66, *n* = 25, *p* = 0.098). The mean value was shortest for the light boxes with uncovered eggs (9.9 min), and greatest for the dark boxes with no visible tit eggs (37.0 min; Fig. 4B).

**Fig. 5:**
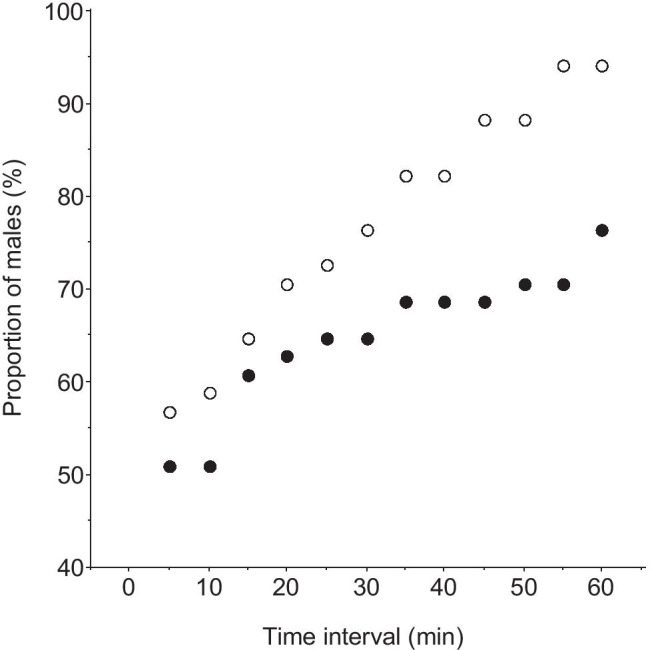
Cumulative distribution for the time interval between the first visit of a nest box by a focal male pied flycatcher and his first entry of the same box. Data for dyads of medium-sized nest boxes with separate values for the nest box with four uncovered great tit eggs (open symbols) and the alternate box with no visible eggs (filled symbols)

The cumulative distribution for the time interval between the first visit of a nest box and the first entry of the same box is shown in Fig. 5 for the medium-sized boxes, with separate values for the nest box with uncovered eggs and the alternate box. At the median time of great tit absence from her nest after the first visit (23 min), 71% of the 51 focal flycatcher males had entered one or more times the nest box with uncovered eggs versus 63% for the alternate box. When 60 min had elapsed, the values were 94% and 76% respectively. We also studied the relative popularity of the two boxes of a dyad after both boxes had been visited at least once. Then, no significant difference occurred in the frequency of visits to the nest box with uncovered eggs versus to the alternative box when comparing medium-sized boxes only (pairwise *t*-test, *t* = 0.79, *n* = 51, *p* = 0.43), nor when including all trials (*t* = 1.42, *n* = 93, *p* = 0.16).

The low number of visiting females precluded detailed analysis of their preferences but the focal male had always entered a box before a female was observed to visit a box in the dyad. The females visited the nest box with exposed eggs sooner than the alternative box (median values of 33 and 40 min from start of trial; Wilcoxon paired signed ranks test, *z* =  − 2.20, *n* = 18, *p* = 0.028).

## Discussion

### Illumination of the cavity interior

Our trials with dyads of boxes showed that prospecting male flycatchers entered a nest box with exposed white eggs after a shorter time, and with fewer visits to the entrance hole compared to boxes with a darker bottom. The relative hesitancy to enter was triggered both when the box contained empty tit nests and when it contained tit eggs covered with a lining. Thus, flycatchers were not wary of egg-covering per se, but appeared to hesitate when the cavity floor was not clearly illuminated. We did not measure light intensities inside the boxes but previous studies have shown that light intensity drops with cavity depth and that color vision is impaired in deep cavities (Wesołowski and Maziarz 2012). Low light probably explains why nestlings of cavity nesting species have light-colored gapes, namely to help the parents locate offspring in dim light (Wiebe and Slagsvold 2009; Wiebe 2010).

The fact that males were less hesitant to investigate light-painted cavities than dark-painted ones supports the idea that interior brightness of the cavity affected the male's perception of danger. The preference for the box of the dyad with luminous eggs on the floor only held for medium-sized boxes. In shallow cavities it might be easy for the flycatcher to see the bottom of the cavity regardless of whether or not the reflective eggs are covered. In deep nest holes, there may be so little light reaching the bottom that even exposed eggs are not visible from the entrance. If the entrance hole is narrow, a perching prospecting bird will also prevent light from entering. The extent of cover within the tit nest increases throughout the laying sequence (Haftorn and Slagsvold 1995) in a way consistent with the usurpation hypothesis because the cavity bottom becomes more illuminated by more eggs. A cavity with a tit nest with eggs visible may easily be identified as a bird´s nest. Perhaps when little light enters a cavity, a nest with only lining visible of hairs, wool and fur can be mis-identified as part of the body of a live mammal.

One may wonder whether visible, uncovered tit eggs might signal cavity occupancy to prospecting birds, causing them to be more reluctant to enter, but we found the opposite. However, our experiments targeted male flycatchers that were displaying at a nest box, so such males may have already recognized there were no tit owners in the vicinity and hence have entered boxes with exposed tit eggs quickly, perceiving them as abandoned. Even if the male flycatcher was aware there were no actively breeding tits, it does not explain his hesitancy to enter dark-painted boxes, suggesting that a lack of illumination in a box per se can indeed cause a wariness to enter.

### Egg covering and fitness consequences for the tit

Typically, prospecting flycatchers seem to avoid investigating active tit nests because being caught by a defending tit owner may be fatal (Merilä and Wiggins 1995; Ahola et al. 2007; Samplonius and Both 2019). For example, in an unmanaged forest in Poland with surplus nest holes, pied flycatchers did not enter cavities with active tit nests (Czeszczewik and Walankiewicz 1999). Similarly, in the present study, we found that flycatchers rarely visited and entered great tit nests during the egg-laying period in contrast to a field site in Finland where male flycatchers entered more frequently and sometimes were killed (Forsman et al. 2018).

During the egg-laying period, female tits must trade-off nest defense with other activities such as foraging and so they often leave the nest, leaving it potentially vulnerable to take-over by other prospecting secondary cavity nesters. Our video-filming showed that the length of absence of the tit was highly variable, from a few minutes to more than an hour, making it difficult for a flycatcher to predict when the tit would return. If a tit must leave its nest for a time, anything it can do to delay the entrance of a prospecting competitor should be beneficial because flycatchers soon perceive themselves as owners of unattended nests and may start to cover the tit eggs with their own nest materials very quickly, which may cause the tit to abandon (Slagsvold 1975). Although covering the eggs with lining did not completely prevent flycatchers from entering, it did delay entrance. For example, after one hour, 94% of flycatcher males had entered a medium-sized trial box with exposed eggs compared to only 76% of males entering when the eggs were covered (Fig. 5).

Although covering the eggs may reduce the risk of usurpation, it also entails costs of collecting additional fur and hair for the egg cover lining and the time to arrange these materials when the female leaves the nest. Costs of obtaining lining material probably depend on its local availability, and the willingness of a female to pay such costs probably depends on a variety of trade-offs such as the need to forage, the risk of predation, and the level of competition for cavities, as shown by a large-scale geographic comparison of egg-covering in tits (Loukola et al. 2020). Great tits add more egg covering when exposed to playback song of pied flycatchers (Loukola et al. 2014a) which supports the idea of facultative investment in a costly behavior. Material used by tits to cover eggs during the egg-laying period may be later incorporated into the nest for increased insulation so it is not necessarily wasted. In great tits, females may collect such materials also during the incubation period (Haftorn and Slagsvold 1995).

After both nest boxes of a dyad had been visited by the focal male flycatcher, he did not visit the nest box with exposed eggs more frequently than the alternative box. Thus, the deterrent of egg lining only appears to be effective during the initial stage of box investigation, before the male knows whether a box contains dangerous tit owners or predators. To reduce the risk to themselves, female pied flycatchers wait for the male to enter first. The few females that we observed also visited the box with exposed eggs significantly sooner than the alternate but the focal male had always entered beforehand, so females may be generally persuaded to enter a cavity that he demonstrates most intensely. Therefore, egg covering may be more influential on the prospecting of male than of female flycatchers. Our trials ended before female flycatchers built nests and hence before they ´chose´ a box so further experiments are needed to test whether cavity contents affect nest preference in the longer term, in natural circumstances.

The lining material we put on top of eggs was only thick enough to just cover them. The egg-cover lining materials may have a longer lasting effect if a felt-like mat of compressed hair and fur makes it difficult for flycatchers to dig their own nest cup into. During filming, we saw some male flycatchers carrying out tit nest materials and some also began to widen the tit nest cup by displacing material without carrying it out. Flycatchers sitting on, and moving around on, existing tit nests has been filmed and interpreted by Loukola et al. (2020) as a way flycatchers assess tit clutch size but we suggest more parsimoniously that the flycatchers are preparing the cavity bottom for their own use. The depth and thickness of any egg-covering material probably interacts with the depth (size) of the cavity to influence its attractiveness to secondary cavity nesters. Cavities that are too shallow raise nest contents close to the cavity entrance and are more at risk of depredation **(**Wesołowski and Maziarz 2012). On the other hand, deep cavities require more new nest materials to raise the nest cup to an optimal height relative to the entrance in a way that balances predation risk with efficiency of feeding nestlings; a shallower nest requires less energy by the parent to enter and leave and also provides more illumination of the offspring (cf. Wesołowski and Maziarz 2012; Fokkema et al. 2018).

### Hypotheses for egg-covering in birds

The hypothesis that egg-covering reduces usurpation assumes there is either intra- or interspecific competition for nest sites and is therefore consistent with some previous findings, namely that tits bring more egg covering materials when exposed to playback of flycatcher song (Loukola et al. 2014a) and egg-covering is more prevalent in regions of Europe with higher densities of flycatchers (Loukola et al. 2020). The hypothesis may also apply to other species. For example, in North America, black-capped chickadees and tufted titmice, may use egg-covering to reduce the risk of nest destruction and cavity usurpation by house wrens (White and Kennedy 1997). Within the geographic range of great tits, other competing cavity-nesters may also reinforce egg-covering behavior, like the Eurasian nuthatch (*Sitta europaea*), the tree sparrow (*Passer montanus*), the European starling (*Sturnus vulgaris*), the wryneck (*Jynx torquill*a), and various species of tits. Nuthatch eggs are also whitish and become buried among the small pieces of bark and wood when the female leaves the nest during egg-laying and incubation (Wesołowski and Rowinski 2004) which may have a similar function as egg-covering in tits. In contrast, flycatchers never cover their eggs perhaps because they start breeding late in the season when the subsequent competition for nest cavities is lower.

A mat of hair and fur may protect the eggs from being damaged during any fights between the cavity owner and an intruder but none of the exposed tit eggs were damaged during the experimental trials, nor have we seen broken tit eggs when finding dead pied flycatchers in great tit nests (TS, unpublished data). This idea of protecting eggs is not mutually exclusive with the usurpation hypothesis nor the hypothesis that the eggs are covered to avoid detection by a predator. Some smaller predators may themselves be at risk of attack or injury by a larger predator when entering an unfamiliar, dark cavity. Therefore, the positive correlation between use of egg covering in great tits and rate of nest predation across Europe (Loukola et al. 2020) may be partly explained by the mechanism of nest illumination as proposed here by the usurpation hypothesis. Blue tits exposed to predator chemical cues in the nest more frequently covered their eggs than birds exposed to an odorous control (Saavedra and Amo 2019). However, more broadly, the nest predation hypothesis proposes that egg covering reduces the likelihood that a nest predator will enter the cavity because of costs of entering without certainty of a reward (Haftorn and Slagsvold 1995; Loukola et al. 2020).

For non-cavity nesting birds, nests are likely to be well-illuminated and so any egg-covering such as that performed by some ducks (Kreisinger and Albrecht 2008) may be better explained by the predation hypothesis and a need to camouflage eggs. However, it is interesting that most open-nesting birds seem to rely on eggshell pigmentation to camouflage eggs unlike cavity-nesters whose eggs generally lack dark pigmentation (Kilner 2006). An interspecific comparison of the use of egg pigmentation versus the use of lining materials to conceal eggs in different environments would help to elucidate the costs and benefits in each case.

The interspecies social information hypothesis proposes that tits cover eggs to prevent prospecting flycatchers from assessing tit clutch size, information that flycatchers purportedly use when selecting their own nest sites (Loukola et al. 2013, 2020). However, a problem with that hypothesis is that the tit clutch must be complete, or nearly so, to provide useful information to the flycatchers and yet many tits cover the eggs with nest material during the laying period, before clutch completion. Furthermore, the behavior proposed by the ´information´ hypothesis is hardly an evolutionary stable strategy. If true, tits with small final clutches should provide information of their low quality to prospecting flycatchers by exposing their eggs and thereby show that the number indeed is low. But then, flycatchers might learn that a tit that tries to conceal its eggs by covering them is one that has a large clutch. Then, the behavior should not be selected because tits that spend time and energy to collect egg covering material will not be able to conceal clutch size information from flycatchers, paying costs with no benefit.

In summary, the new hypothesis that we propose, the usurpation hypothesis, explains that cavity nesting birds cover their eggs during the egg-laying period to reduce the cost of defending the nest site and thereby prevent usurpation of the cavity by other birds. Our experiments with nest boxes supported the prediction that prospecting flycatchers hesitated to enter dark cavities with dark floors compared to boxes with exposed, reflective eggs. Extrapolating the results obtained from nest boxes to natural cavities must be done cautiously (Wesołowski 2011). The managed forests of our study site had few natural cavities but there may be little competition for cavities in some old, unmanaged forests (Czeszczewik and Walankiewicz 1999; Wiebe 2011). Therefore, the usurpation hypothesis should be studied in other forest types and in relation to the depths of natural cavities.

## Data Availability

https://doi.org/10.5061/dryad.cz8w9gj35
